# *Trichinella spiralis* paramyosin activates mouse bone marrow-derived dendritic cells and induces regulatory T cells

**DOI:** 10.1186/s13071-016-1857-y

**Published:** 2016-11-04

**Authors:** Kai Guo, Ximeng Sun, Yuan Gu, Zixia Wang, Jingjing Huang, Xinping Zhu

**Affiliations:** 1 Department of Medical Microbiology and Parasitology, School of Basic Medical Sciences, Capital Medical University, Beijing, 100069 China; 2 Research Centre of Microbiome, Capital Medical University, Beijing, 100069 China

**Keywords:** Dendritic cells, *Trichinella spiralis*, Paramyosin, Regulatory T cells

## Abstract

**Background:**

Dendritic cells (DCs) are antigen-presenting cells that regulate T cell responses for many infectious diseases. The tissue-dwelling nematode *Trichinella spiralis* expresses paramyosin (*Ts*Pmy) not only as a structural protein but also as an immunomodulator to alleviate complement attack by binding to some host complement components. Whether *Ts*Pmy is involved in other immunomodulatory pathway and how *Ts*Pmy interacts with host DCs is still unknown.

**Methods:**

Mouse bone marrow-derived DCs were incubated with recombinant *Ts*Pmy (r*Ts*Pmy) for activation. Maturation of DC was determined by the expression of surface markers CD40, CD80, CD86 and MHCII. The r*Ts*Pmy-pulsed DCs were co-incubated with *T. spiralis*-sensitized or naïve mouse CD4^+^ T cells to observe their activation on T cells and polarizing regulatory T cells using flow cytometry. Cytokines were measured by enzyme-linked immunosorbent assays (ELISA).

**Results:**

*Ts*Pmy was able to activate mouse bone marrow-derived DCs to semi-mature status characterized by expressing surface CD40 and CD86, but not CD80 and MHCII. The semi-mature *Ts*Pmy-pulsed DCs were able to stimulate *T. spiralis*-sensitized CD4^+^ T cells to proliferate. Incubation of *Ts*Pmy-pulsed DCs with naïve CD4^+^ splenocytes polarized the latter to CD4^+^CD25^+^Foxp3^+^ regulatory T cells. However, mice immunized with r*Ts*Pmy only induce the CD4^+^CD25^−^Foxp3^+^ T cell population, associated with high level of IL-10, TGF-β and IL-17A.

**Conclusions:**

During *T. spiralis* infection, *Ts*Pmy plays an important role in modulating the host immune system by stimulating DCs to differentiate the CD4^+^ T cells to regulatory T cells, in addition to binding to components of the host complement cascade, as survival strategies to live in host.

## Background

Trichinellosis is a serious zoonotic parasitic disease caused by the infection of *Trichinella spiralis* through ingestion of meat contaminated with infective larvae*.* It is estimated that more than 11 million people could be infected with *T. spiralis* worldwide [1] and heavy infection can even causes death [2]. *Trichinella spiralis* is a tissue-dwelling parasitic nematode. During *T. spiralis* infection, the entire life-cycle is completed within the same host. After being ingested, the infective muscle larvae develop to adult worms in the host intestine. The newborn larvae are released from sexually-mature adult worms and soon migrate to skeletal muscles to form encysted muscle larvae that may live for several years and cause a chronic infection [3]. How the *Trichinella* parasite maintains the chronic infection within the host without being recognized and attacked by the host’s immune system remains unknown [4]. Understanding the mechanism underlying the immune evasion would greatly benefit the design of preventive/therapeutic vaccines or drugs to control the infection.

Paramyosin is not only a fibrillar protein exclusively found in invertebrates, but also a functional protein expressed on the surface of many helminths [5–7] that plays an important role as an immunomodulatory molecule to defend against host immune attack [8–10]. Paramyosin of *T. spiralis* (*Ts*Pmy) was cloned from the adult *T. spiralis* in a previous study [11]. Subsequent studies have identified that *Ts*Pmy binds to host complement components C8, C9 and C1q that interferes with the forming of complement membrane attack complex and protects parasite from being attacked by the host innate immune system [12–15]. Partial protective immunity against *T. spiralis* larval challenge was determined in BALB/c mice immunized with recombinant *Ts*Pmy (r*Ts*Pmy) [16] and protective epitopes [17–19] or through RNAi [20]. Except for interfering with host complement system, whether *Ts*Pmy is involved in other immunomodulatory function is unknown.

Dendritic cells (DCs) are antigen presenting cells that play a pivotal role in the control and modulation of immune responses by initiating T cell responses and producing cytokines and other molecules that regulate adaptive immunity [21]. How *Ts*Pmy interacts with DC and subsequently impacts DC activation and function during *T. spiralis* infection is not understood.

In this study, we investigated the roles of *Ts*Pmy on DCs maturation and subsequent T cell polarization. The study herein demonstrated that *Ts*Pmy could activate bone marrow-derived mouse DCs and consequently promote the differentiation of CD4^+^ T cells to regulatory T cells (Tregs). The induction of Tregs by *Ts*Pmy through activated DCs during *T. spiralis* infection may inhibit the host immune response and play an important role in the survival of *T. spiralis* in infected host.

## Methods

### Experimental animals

Specific pathogen-free 6–8 week-old female BALB/c mice were purchased from the Laboratory Animal Services Center of the Capital Medical University (Beijing, China) and housed under specific pathogen-free conditions with humidity and temperature controlled (temperature of 20 ± 2 °C; humidity of 70 ± 10 %). All animal protocols and husbandry were approved by Capital Medical University Institutional Animal Care and Use Committee (IACUC).

### Parasites and experimental infection

The ISS 533 strain of *T. spiralis* was maintained in female ICR mice. Muscle larvae (ML) were received from the muscles of infected mice by previously described method of modified pepsin-hydrochloric acid digestion [17]. BALB/c mice were infected with 400 infective *T. spiralis* ML by oral gavage and immunized with recombinant *Ts*Pmy (r*Ts*Pmy) as described below.

### Antigen preparation

The recombinant *Ts*Pmy with a His-tag at C-terminus was expressed in Baculovirus/insect cell Sf9 (Invitrogen, Carlsbad, CA, USA) and purified with Ni-affinity chromatography (Qiagen, Valencia, CA, USA). Lipopolysaccharide (LPS) (Sigma-Aldrich, St. Louis, MO, USA) was used as a positive control for immune response. Sf9 cell lysis proteins were used as non-relevant proteins control. All antigens were stored at -80 °C.

### Generation of dendritic cells

DCs were generated from mouse bone marrow cells as described [22]. Briefly, bone marrow cells were obtained from BALB/c mice and cultivated in RPMI 1640 medium (Hyclone, Logan, UT, USA) supplemented with 10 % foetal bovine serum (FBS; Thermo Fisher, Life Technologies, Carlsbad, CA, USA) and penicillin/streptomycin at 37 °C, 5 % CO_2_ for 3 h. After removing the suspended cells, the remaining adherent cells were cultured in RPMI 1640 medium containing growth factors of 10 ng/ml recombinant GM-CSF and 2 ng/ml IL-4 (Prospec, Rehovot, Israel) and 10 % FBS for 6 days with replenishment of the same culture medium on Day 3 and Day 5. The immature DCs were harvested on Day 6 for further experiments.

### In vitro DC activation

The immature DCs produced above were stimulated with r*Ts*Pmy (10 μg/ml), LPS (2 μg/ml) or PBS respectively in vitro for 48 h. The stimulated cells were stained with APC-conjugated monoclonal antibody (mAb) to CD11c, the major marker of mature DCs [23], and PE-conjugated mAbs to major histocompatibility complex II (MHCII), CD40, CD80 or CD86 respectively (BD Biosciences, San Jose, CA, USA). The cytokine levels in the culture supernatants were determined by the corresponding enzyme-linked immunosorbent assay (ELISA) kits according to the manufacturer’s instructions (Dakewe Biotech, Shenzhen, China).

### Co-incubation of r*Ts*Pmy pulsed DCs with *T. spiralis*-sensitized CD4^+^ T cells

To determine whether r*Ts*Pmy-pulsed DCs could activate *T. spiralis*-sensitized CD4^+^ T cells, the DCs pulsed with r*Ts*Pmy for 72 h, and then were co-cultivated with *T. spiralis*-sensitized CD4^+^ T cells, the *T. spiralis*-sensitized CD4^+^ T cells were obtained from the spleens of BALB/c mice infected with 400 *T. spiralis* ML for 60 days using magnetic-activated cell sorting (MACS) with a mouse CD4^+^ T cell isolation kit (Miltenyi Biotec, Bergisch Gladbach, Germany). A total of 5 × 10^4^ or 2.5 × 10^4^ DCs were plated in each well of round-bottom 96-well plates and then co-cultivated with 5 × 10^5^ *T. spiralis*-sensitized CD4^+^ T cells stained with 5-and 6-carboxyfluorescein diacetate succinimidyl ester (CFSE) (eBioscience, San Diego, CA, USA), in the presence of 5 μg/ml Concavalin-A (Con-A) (Sigma-Aldrich, St. Louis, MO, USA) which is a nonspecific stimulator for mouse T cells. Subsequently, the proliferation of T cells was measured by fluorescence-activated cell sorting (FACS).

To determine the cytokine production, 5 × 10^4^ DCs were plated in each well of round-bottom 96-well plates and co-incubated with 5 × 10^5^ *T. spiralis*-sensitized CD4^+^ T cells for 36 h, then supernatants were collected and cytokines measured by ELISA as described above.

### r*Ts*Pmy pulsed DCs/naïve CD4^+^ T cells co-incubation

To assess the ability of r*Ts*Pmy-pulsed DCs on naïve T cell polarization, DCs were stimulated with r*Ts*Pmy for 72 h, and the naïve CD4^+^ T cells were isolated from the spleens of healthy BALB/c mice by MACS using mouse CD4^+^ T cell isolation kit. Total 5 × 10^4^ DCs were plated in each well of round-bottom 96-well plates and co-incubated with 5 × 10^5^ naïve CD4^+^ T cells. The co-incubated DCs/naïve T cells were stimulated with 5 μg/ml plate-bound anti-CD3/anti-CD28 (Peprotech, NJ, USA) which delivers signal one and a costimulatory signal two without leading to early cell death for proliferated cells [24]. The co-incubation was continued at 37 °C for 36 h and cells were recovered for detecting the percentage of CD4^+^CD25^+^Foxp3^+^ T cells. Meanwhile, the co-incubation supernatants were collected for detection of cytokines level by ELISA as described above.

### T cell response primed by r*Ts*Pmy in vivo

BALB/c mice were divided into 3 groups with 4 mice each, and each group was immunized intraperitoneally with 100 μg of r*Ts*Pmy or the same amount of Sf9 insect cell protein as a non-relevant protein control twice at 2 weeks intervals. Another group of 4 mice were given PBS only. Fourteen days after the final immunization, all mice were sacrificed and the splenocytes were harvested for the analysis of cytokine production and the presence of Th17 cells and Tregs.

For FACS analysis of Th17 cells, the harvested splenocytes were stimulated with 25 ng/ml phorbol-12-myristate-13-acetate (PMA, Sigma-Aldrich, St. Louis, MO, USA), 1 μg/ml ionomycin (Sigma-Aldrich, St. Louis, MO, USA) and 0.66 μl/ml Golgistop™ (BD Biosciences, San Jose, CA, USA) for 6 h before cell staining with anti-IL-17A-PE-Cyanine7 (eBioscience, San Diego, CA, USA). The culture supernatants were recovered for measuring cytokine release as described above. For detection of Tregs, the harvested splenocytes were directly stained with Mouse Regulatory T Cell Staining Kit #1 according to the manufacturer’s instructions (eBioscience, San Diego, CA, USA).

### Statistical analysis

GraphPad Prism version 6 software (San Diego, CA, USA) was used to analyze statistical data. The results are presented as mean ± standard deviation. Statistical significance was determined by one-way ANOVA with Dunnett or Tukey’s *post-hoc* analysis; *P* < 0.05 was considered statistically significant.

## Results

### Semi-maturation of DCs after r*Ts*Pmy stimulation

**Fig. 1:**
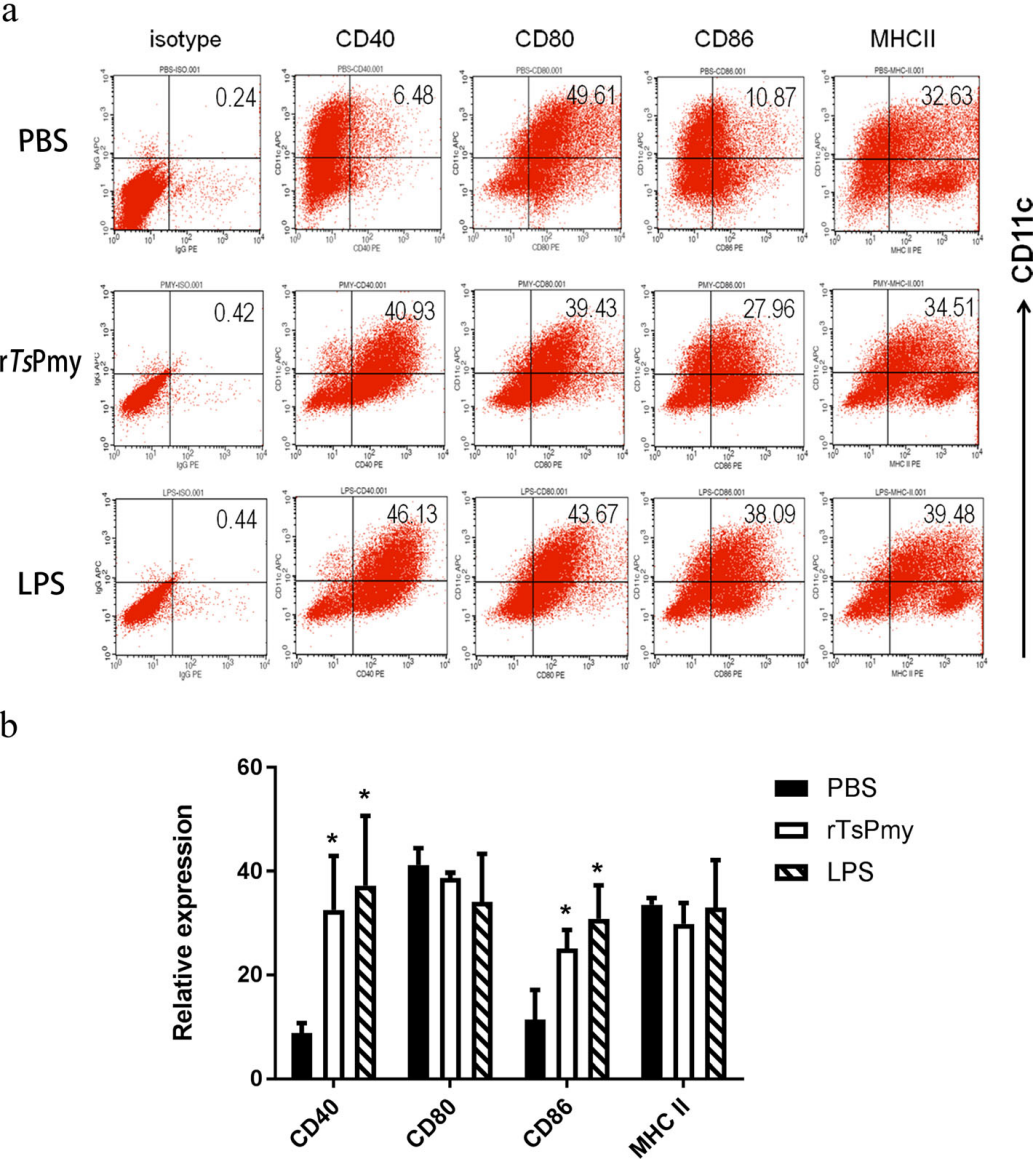
Expression of surface markers on DCs pulsed with r*Ts*Pmy. DCs were pulsed with PBS, r*Ts*Pmy or LPS for 48 h. The surface markers CD40, CD80, CD86 and MHCII were sorted by FACS (**a**). The percentage of each surface marker expression on DCs stimulated with the indicated antigens (**b**). Data represent means ± standard deviations of the results from three individual experiments. *P < 0.05 compared to PBS control

FACS data demonstrated that both r*Ts*Pmy and LPS (positive control) significantly upregulated the expression of CD40 and CD86 on stimulated CD11c^+^ DCs compared to PBS control (r*Ts*Pmy *vs* PBS: CD40, *t*_*D*(6)_ = 2.963, *P* = 0.044; CD86, *t*_*D*(6)_ = 3.106, *P* = 0.037; LPS *vs* PBS, CD40, *t*_*D*(6)_ = 3.547, *P* = 0.021; CD86, *t*_*D*(6)_ = 4.213, *P* = 0.01) (Fig. 1). However, r*Ts*Pmy and LPS did not stimulate the expression of CD80 and MHCII on CD11c^+^ DCs (CD80, *F*_(2,6)_ = 1.209, *P* = 0.362; MHCII, *F*_(2,6)_ = 0.6119, *P* = 0.573). These results indicate that r*Ts*Pmy was able to stimulate the BMDCs to semi-mature status.

### Detection of cytokine production of DCs response to r*Ts*Pmy

**Fig. 2:**
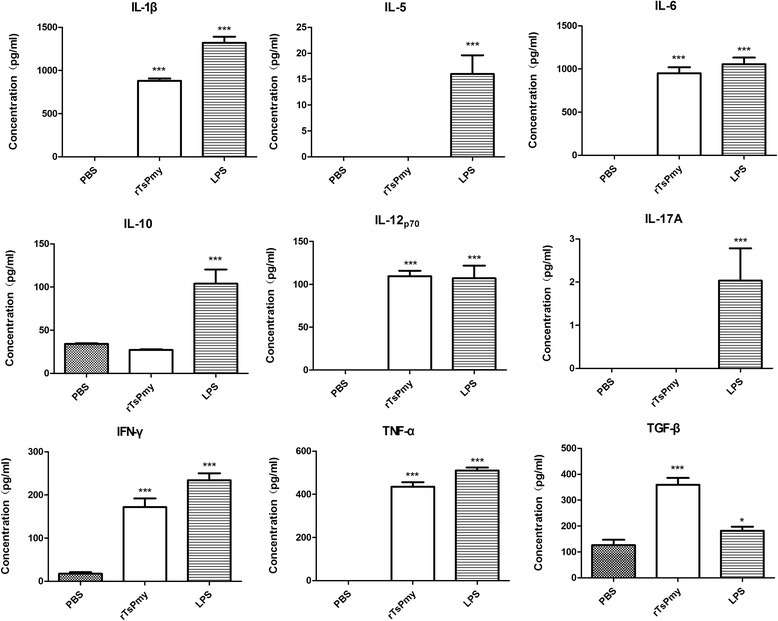
Cytokine production by DCs stimulated with r*Ts*Pmy. DCs were cultured with r*Ts*Pmy, LPS or PBS for 48 h. Culture supernatants were then collected and the levels of different cytokines were determined by ELISA. Results are presented as the mean ± standard deviation from three individual experiments. *P < 0.05; ***P < 0.001 compared to PBS control

To further investigate if r*Ts*Pmy stimulates DCs to secrete Th1, Th2, Th17 and regulatory cytokines, IL-1β, IL-5, IL-6, IL-10, IL-17A, IL-12p_70_, IFN-γ, TNF-α and TGF-β were detected in culture supernatants of antigen-stimulated DCs. Compared to the PBS control, IL-1β, IL-6, IL-12p_70_, IFN-γ, TNF-α and TGF-β were significantly elevated following r*Ts*Pmy stimulation (IL-1β, *t*_*D*(6)_ = 24.95, *P* < 0.001; IL-6, *t*_*D*(6)_ = 27.28, *P* < 0.001; IL-12p_70_, *t*_*D*(6)_ = 15.02, *P* < 0.001; IFN-γ, *t*_*D*(6)_ = 12.55, *P* < 0.001; TNF-α, *t*_*D*(6)_ = 51.19, *P* < 0.001; TGF-β, *t*_*D*(6)_ = 14.13, *P* < 0.001), indicating a mixed Th1/Th2/Treg responses. There was no change in the secretion of IL-5, IL-10 and IL-17A in the supernatants of r*Ts*Pmy-stimulated DCs (Fig. 2). As a positive control, LPS stimulated secretion of all detected cytokines.

### r*Ts*Pmy-pulsed DCs activate *T. spiralis*-sensitized CD4^+^ T cells

**Fig. 3:**
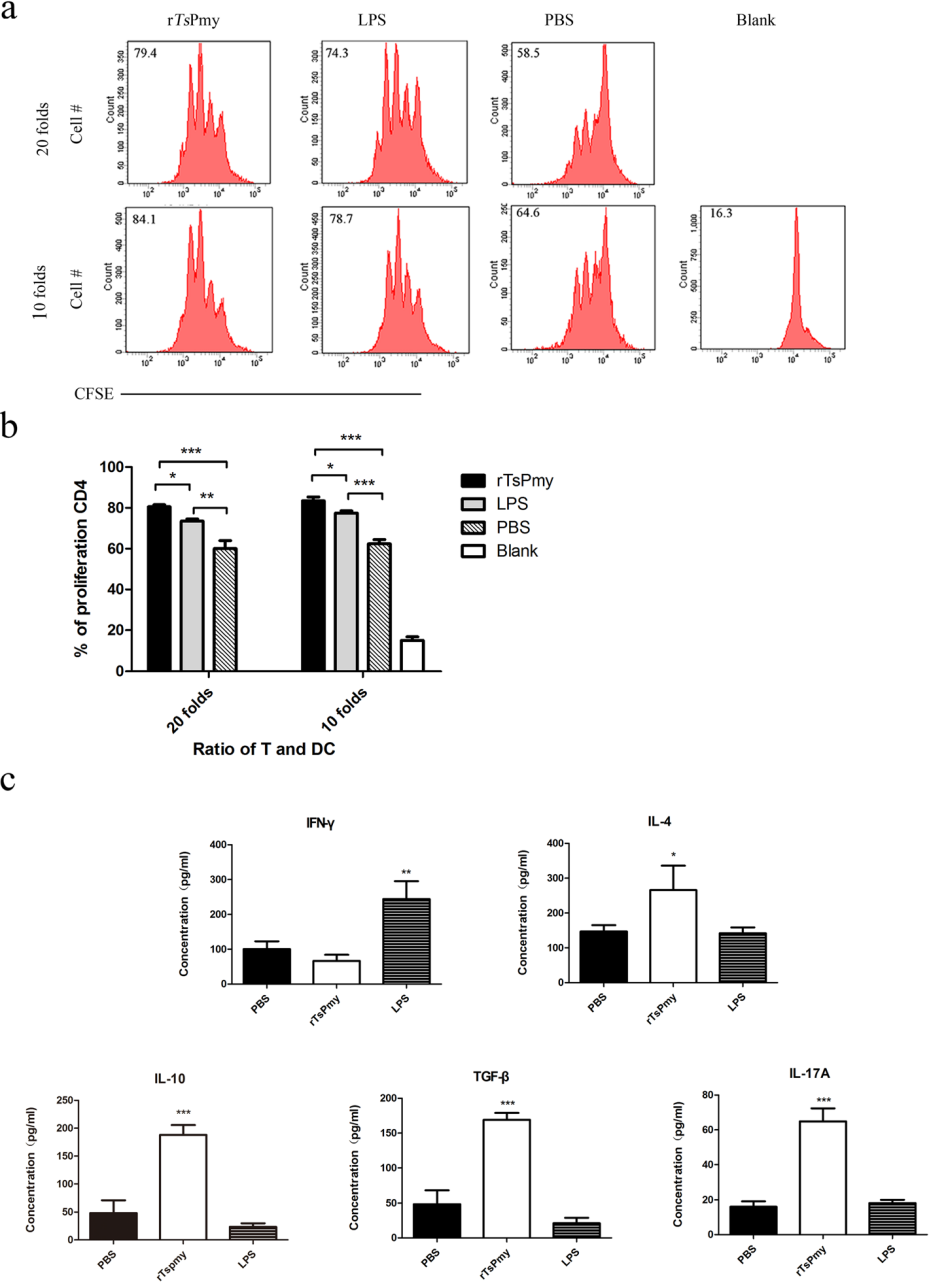
Proliferation and cytokines secretion of *T. spiralis*-sensitized CD4^+^ T cells co-incubated with r*Ts*Pmy-pulsed DCs. To assess whether r*Ts*Pmy-pulsed DCs enable to stimulate *T. spiralis*-sensitized CD4^+^ T cells, the CD4^+^ T cells from splenocytes of *T. spiralis* infected BALB/c mice were incubated with r*Ts*Pmy-, LPS- or PBS-treated DCs and the proliferation of co-incubated T cells were determined by CFSE labeling and FACS (**a**, **b**). The levels of IFN-γ, IL-4, IL-10, TGF-β and IL-17A in the culture supernatants were measured by ELISA (**c**). Data are presented as the mean ± standard deviation from three individual experiments. *P < 0.05; **P < 0.01; ***P < 0.001

To assess whether r*Ts*Pmy-pulsed DCs could activate *T. spiralis*-sensitized CD4^+^ T cells, DCs pulsed with r*Ts*Pmy for 72 h were co-cultivated with *T. spiralis*-sensitized CD4^+^ T cells from *T. spiralis*-infected mice for 72 h in the presence of Con-A. FACS results revealed that *T. spiralis*-sensitized CD4^+^ T cells were significantly induced by r*Ts*Pmy-pulsed DCs with a significantly higher proliferation rate compared to PBS incubated DCs (20 folds: *q*_(6)_ = 14.83, *P* < 0.001; 10 folds: *q*_(6)_ = 20.9, *P* < 0.001) (Fig. 3a, b). LPS-pulsed DCs also boosted T cell proliferation at a lower rate than that induced by r*Ts*Pmy-pulsed DCs.

Cytokine profiling demonstrated that *T. spiralis*-sensitized CD4^+^ T cells secreted higher level of IL-4, IL-10, TGF-β and IL-17A when being incubated with r*Ts*Pmy-pulsed DCs compared to cells incubated with LPS or PBS treated DCs (r*Ts*Pmy *vs* PBS: IL-4, *t*_*D*(6)_ = 3.367, *P* = 0.027; IL-10, *t*_*D*(6)_ = 9.988, *P* < 0.001; TGF-β, *t*_*D*(6)_ = 10.92, *P* < 0.001; IL-17A, *t*_*D*(6)_ = 13.38, *P* < 0.001) (Fig. 3c). However, r*Ts*Pmy-pulsed DCs did not stimulate the secretion of IFN-γ in *T. spiralis*-sensitized T cells (*t*_*D*(6)_ = 1.227, *P* = 0.417). The cytokine profile results support that helminth infections generally polarize the T cell response towards Th2, while IL-10 and TGF-β might suppress Th1 response and therefore inhibit the production of IFN-γ. The results of proliferation and cytokine profiling revealed that *Ts*Pmy-pulsed DCs are able to activate T cells or boost the memory T cells with Th2 and Treg-related cytokine responses. LPS-stimulated DCs only induced some level of IFN-γ in CD4^+^ *T. spiralis*-sensitized T cells.

### r*Ts*Pmy-pulsed DCs induces naïve T cells to polarize to Tregs

**Fig. 4:**
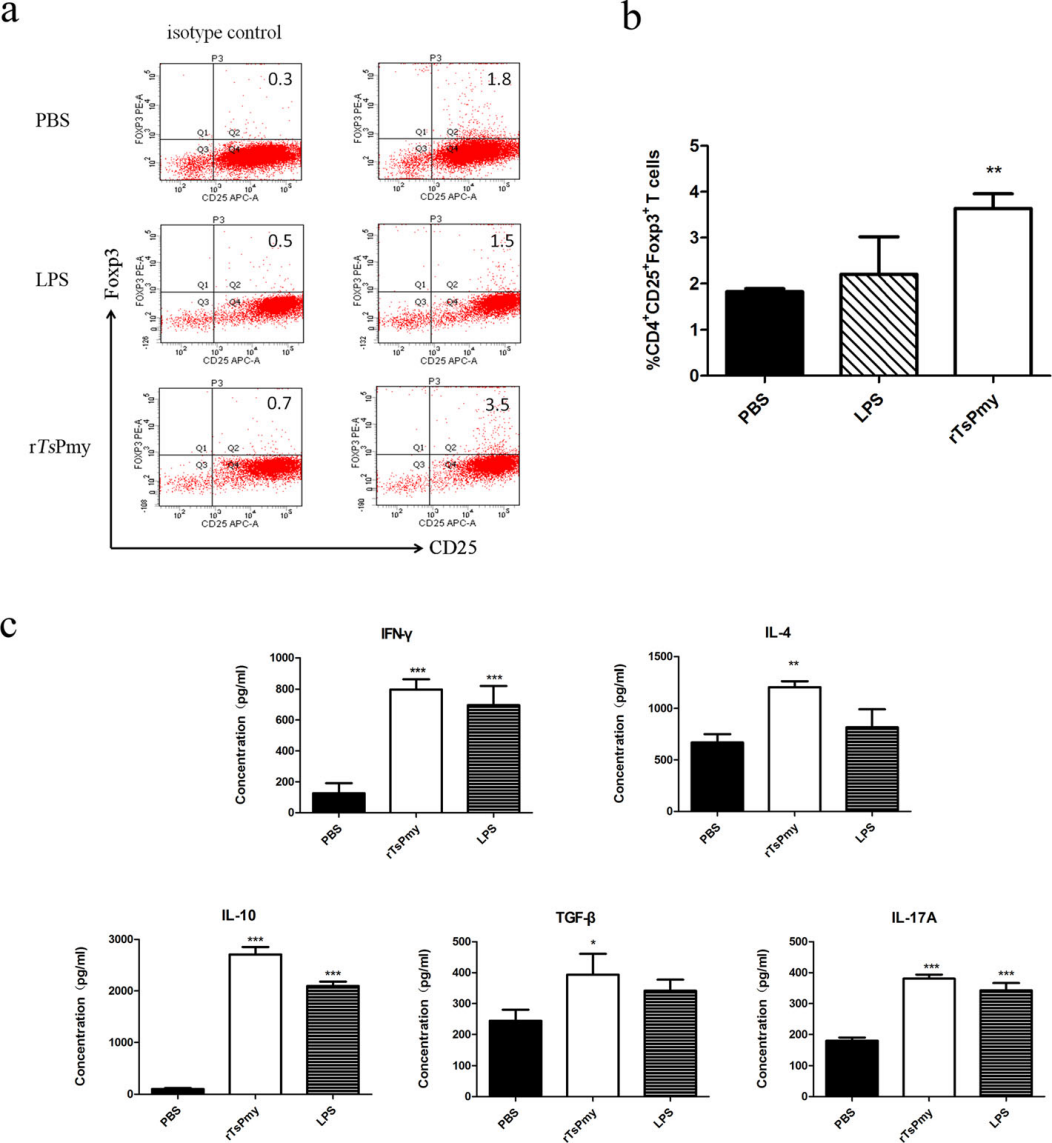
Induction of CD4^+^CD25^+^ Foxp3^+^ T cell population and Treg cytokines in naïve T cells when incubated with r*Ts*Pmy-pulsed DCs. The splenocytes isolated from naïve BALB/c mice were incubated with r*Ts*Pmy-, LPS- or PBS-treated DCs for 36 h, then labeled with anti-CD4-FITC and anti-CD25-APC and anti-Foxp3-PE for FACS plot analysis (**a**). The percentage of CD4^+^CD25^+^Foxp3^+^ cells in CD4^+^ T cell population was showed in **b**. **c** Cytokine profile of naïve CD4^+^ T cells incubated with r*Ts*Pmy-, LPS- or PBS-treated DCs. The co-incubation supernatants were recovered and measured for secretions of IFN-γ, IL-4, IL-10, TGF-β and IL-17A by ELISA. Data are presented as the mean ± standard deviation from three individual experiments. *P < 0.05; **P < 0.01; ***P < 0.001 compared to PBS control

In order to determine whether r*Ts*Pmy-pulsed DCs induce naïve CD4^+^ T cell polarization, the r*Ts*Pmy-treated DCs were incubated with naïve T cells isolated from spleens of normal BALB/c mice for 36 h. The FACS results demonstrated that the CD4^+^CD25^+^Foxp3^+^ T cell population was significantly elevated in naïve T cells co-cultivated with r*Ts*Pmy-pulsed DCs compared to PBS-treated DCs (*t*_*D*(6)_ = 4.333, *P* = 0.009) (Fig. 4a, b), while LPS-pulsed DCs did not obviously affected the population of CD4^+^CD25^+^Foxp3^+^ T cells (*t*_*D*(6)_ = 0.8826, *P* = 0.608).

In parallel, the culture supernatants were measured for IFN-γ,IL-4, IL-10, TGF-β and IL-17A secretion. The results revealed that r*Ts*Pmy-pulsed DCs not only induced naïve T cells to secrete cytokine IFN-γ (Th1), IL-4 (Th2) and IL-17A (Th17) (IFN-γ, *t*_*D*(6)_ = 11.23, *P* < 0.001; IL-17A, *t*_*D*(6)_ = 14.68, *P* < 0.001; IL-4, *t*_*D*(6)_ = 6.626, *P* = 0.001), but also stimulated high levels of cytokines IL-10 and TGF-β secreted mostly by Tregs (IL-10, *t*_*D*(6)_ = 32.71, *P* < 0.001; TGF-β, *t*_*D*(6)_ = 4.211, *P* = 0.01) (Fig. 4c), which is consistent with the increase of Treg population observed by FACS. LPS-pulsed DCs also stimulated naïve T cells to secrete IFN-γ, IL-10 and IL-17A, but the level was not as high as that induced by r*Ts*Pmy-pulsed DCs.

### r*Ts*Pmy induces Treg in immunized mice

**Fig. 5:**
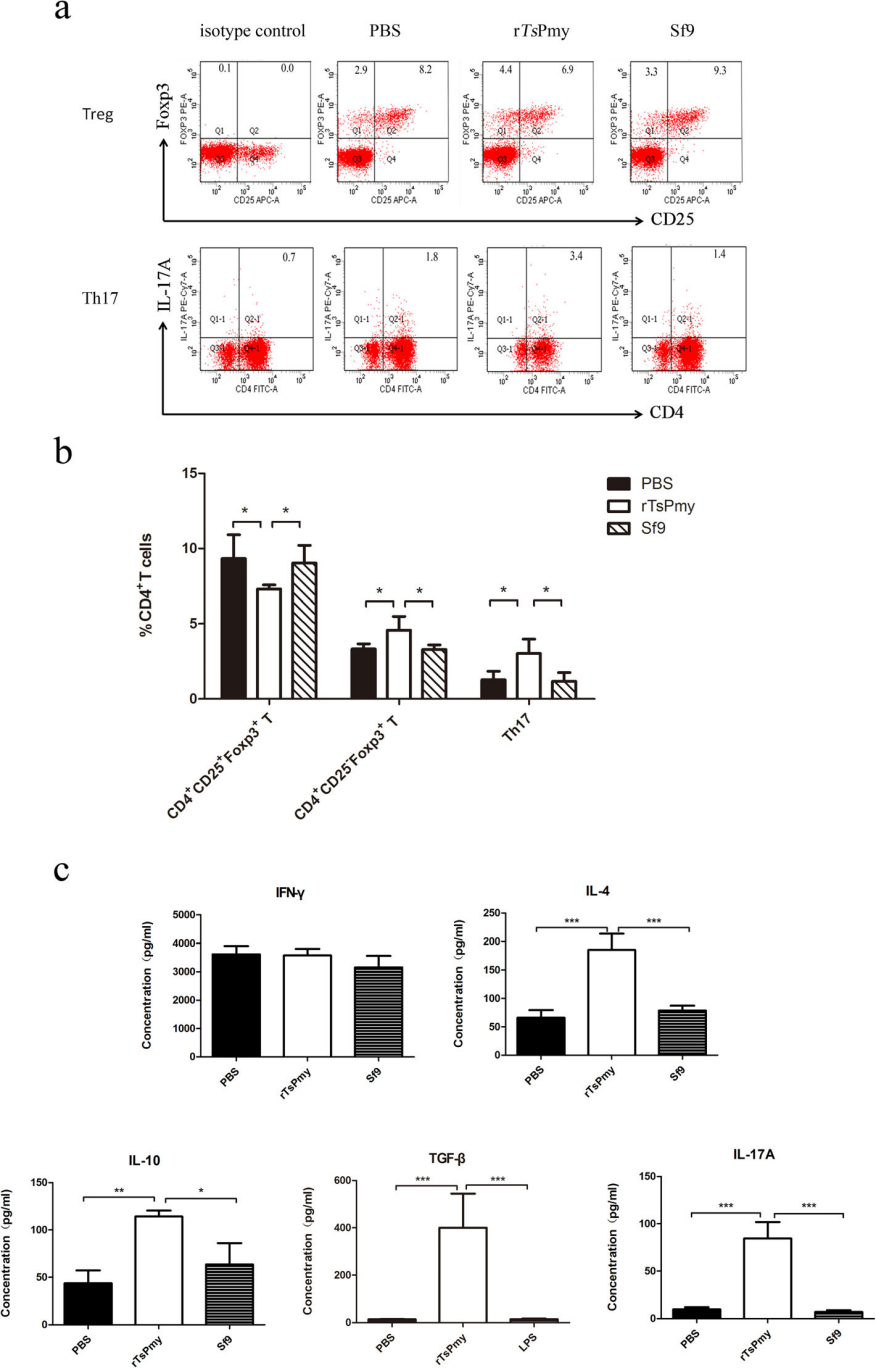
Tregs and Th17 cell differentiation and cytokines production in splenocytes of mice immunized with r*Ts*Pmy. Splenocytes were isolated from mice immunized with r*Ts*Pmy or Sf9 protein and sorted for CD4, CD25, Foxp3 and Th17 by FACS (**a**, **b**). The different cytokines in splenocyte culture were determined by ELISA (**c**). Data (means ± standard deviations) are representative of three independent experiments. *P < 0.05; **P <0.01; ***P <0.001 compared to PBS or Sf9 control as indicated

To confirm if *Ts*Pmy enables to induce Treg in vivo, BALB/c mice were immunized with r*Ts*Pmy and the CD4^+^CD25^+^Foxp3^+^ T cells were sorted from splenocytes of immunized mice. The FACS results demonstrated that r*Ts*Pmy immunization did not increase the population of CD4^+^CD25^+^Foxp3^+^ T cells compared to PBS or non-relevant Sf9 protein control groups, however, CD4^+^CD25^−^Foxp3^+^ T cells were upregulated (r*Ts*Pmy *vs* PBS: *t*_*D*(9)_ = 3.005, *P* = 0.027). Interestingly, the Th17 cells were also upregulated in r*Ts*Pmy immunized mice (r*Ts*Pmy *vs* PBS: *t*_*D*(9)_ = 3.402, *P* = 0.014) (Fig. 5a, b). As we know Th17 cells are able to convert T cells into Tregs in mesenchymal stem cell-mediated allograft survival [25].

To further investigate the cytokine profile secreted by splenocytes of r*Ts*Pmy immunized mice, splenocytes from immunized mice were isolated and stimulated with PMA/ionomycin. The different cytokine level in the culture supernatants was detected by ELISA. Results showed IL-4, IL-10, TGF-β and IL-17A levels were significantly elevated in cultures of splenocytes from mice immunized with r*Ts*Pmy compared to PBS (IL-4, *t*_*D*(9)_ = 7.482, *P* < 0.001; IL-10, *t*_*D*(6)_ = 5.507, *P* = 0.003; TGF-β, *t*_*D*(9)_ = 6.55, *P* < 0.001; IL-17A, *t*_*D*(10)_ = 10.01, *P* < 0.001) or non-relevant protein (Sf9) injection control groups (IL-4, *t*_*D*(9)_ = 6.696, *P* < 0.001; IL-10, *t*_*D*(6)_ = 3.964, *P* = 0.013; TGF-β, *t*_*D*(9)_ = 6.554, *P* < 0.001; IL-17A, *t*_*D*(10)_ = 10.38, *P* < 0.001) (Fig. 5c). There was no change in IFN-γ level (*F*_(2,7)_ = 2.162, *P* = 0.186). The secretions of regulatory cytokine IL-10, TGF-β are consistent with the differentiation of CD4^+^CD25^−^Foxp3^+^ Tregs.

## Discussion

During pathogen infections, DCs play a critical role in the induction and orchestration of immune responses. The infection itself induces DC activity and maturation through various families of pattern recognition receptors (PRRs) such as Toll-like receptors [26, 27], and produces different cytokines to prime distinct types of adaptive immune responses [28]. Therefore, DC responses are crucial to control and eliminate the invading pathogens during infection [29]. However, the specific mechanism during interaction between DC and helminthic antigens, especially in the role of helminth immune evasion, are still not quite understood.

We have identified that *Ts*Pmy is a strong immunomodulator by interfering with host complement functions as a strategy to evade host innate immune attack in our previous studies [12–15]. In this study, we found that r*Ts*Pmy enabled to stimulate mouse bone marrow-derived DCs to express CD40, CD86, but not CD80 and MHCII, on the surface of CD11c^+^ DCs. r*Ts*Pmy-pusled DCs secreted high level of IL-1β, IL-6, IL-12p70, IFN-γ, TNF-α and TGF-β, but not for IL-5, IL-10 and IL-17A, indicating r*Ts*Pmy induces DCs to a semi-mature status in vitro to secrete a mix Th1/Th2/Treg response in cytokine expression. Our results are consistent with other studies that showed parasitic helminth antigens activated DCs to incomplete maturation [22, 30, 31].

When incubating with *T. spiralis*-infected mouse splenocytes, the r*Ts*Pmy-pulsed DCs stimulated the *T. spiralis*-infected mouse CD4^+^ cells to proliferate (Fig. 3a, b), but did not strongerly stimulate the naïve mouse CD4^+^ cells to proliferate (data not shown), indicating r*Ts*Pmy-activated DCs was able to present *Ts*Pmy antigen to T cells previously exposed to *T. spiralis* infection to activate the *Ts*Pmy memory cells. The cytokine profile also confirmed that r*Ts*Pmy-pulsed DCs stimulated *T. spiralis*-infected mouse CD4^+^ cells to secrete IL-4, IL-10, TGF-β and IL-17A, but not IFN-γ, consistent with the Th2-skewed immune response induced by helminth infections [32].

Evasion of host adaptive immunity is key strategy for the survival of parasites in the hostile environment within the host [33, 34]. Many studies have demonstrated both helminths and protozoans create more permissive environments for surviving in hosts by interfering with DCs activity [29, 35]. Helminths and their products have been shown to suppress immune response of the host by inducing a regulatory network. DCs play a crucial role in this regulatory network, as they can regulate T cell-mediated effector responses by generating anti-inflammatory cytokines that can lead to induction of regulatory T cells [36] and promote parasite immune escape by inhibiting parasite-specific immune responses [37]. In order to determine if *Ts*Pmy possesses the same immunomodulatory ability to induce tolerogenic DCs so as to stimulate the host T cell regulatory network, the r*Ts*Pmy was incubated with DCs and the r*Ts*Pmy-pulsed DCs were co-incubated with naïve mouse CD4^+^ T cells. The results showed that *Ts*Pmy-pulsed DCs enabled to induce CD4^+^CD25^+^Foxp3^+^ Treg cells in vitro associated with higher level of IL-10 and TGF-β, the cytokines mostly secreted by Tregs [38]. Our results confirmed that *Ts*Pmy was able to stimulate tolerogenic DCs that subsequently induce Treg cells to modulate host immune response, possibly through signal passage from DC to T cells or *Ts*Pmy-pulsed DC-secreted Th1/Th2/Treg cytokines (Fig. 2). Even though r*Ts*Pmy only induced bone marrow-derived DCs to a semi-mature status, it did not affect their abilities to induce Treg cells, confirming that semi-mature DCs also induce tolerance [39, 40]. Our results are consistent with other investigations that showed *T. spiralis* excretory-secretory antigen-stimulated dendritic cells alleviated experimental autoimmune encephalomyelitis or DSS-induced colitis through inducing Treg that increased the secretion of IL-4, IL-10 and TGF-β [4, 41, 42]. However, r*Ts*Pmy immunization only induced CD4^+^CD25^−^Foxp3^+^ T cells, not CD4^+^CD25^+^Foxp3^+^ T cells, in immunized mice. There could be an explanation that experiments represent the situation simplified in vitro, differ from originally existing in live infection. It is possible that r*Ts*Pmy stimulates the Tregs through multiple channels in vivo except for inducing tolerogenic DCs (such as CTLA-4, TGF-β, IL-10, and GITR). Naïve T cells can be converted to a Treg phenotype by culture with CTLA-4-Ig [43]. IL-6 can convert CD4^+^CD25^+^Foxp3^+^ Tregs but not CD4^+^CD25^−^Foxp3^+^ Tregs to Th17 cells [44]. The reasons why CD4^+^CD25^−^Foxp3^+^ Tregs, not CD4^+^CD25^+^Foxp3^+^ Tregs were induced with r*Ts*Pmy immunization need to be further studied. Nevertheless, Foxp3 expression, rather than CD25 expression is essential for Treg’s activity [45]. CD4^+^CD25^−^Foxp3^+^ T cells also showed suppressive activity [45, 46]. Actually, during *T. spiralis* chronic infection, CD4^+^CD25^−^ effector T cells control inflammation, rather than CD4^+^CD25^+^ Tregs [47]. It was an interesting finding that r*Ts*Pmy immunization in vivo generated CD4^+^CD25^−^Foxp3^+^ Tregs that is different from in vitro stimulation of CD4^+^CD25^+^Foxp3^+^ Tregs via inducing tolerogenic DCs.

In addition, in this study we identified that r*Ts*Pmy-pulsed DCs induced *T. spiralis*-infected mouse CD4^+^ or naïve CD4^+^ T cells to produce high level of IL-17A. Mice immunized with r*Ts*Pmy in vivo also induced the generation of Th17 cells. Even though Th17 cells have been considered to be pro-inflammatory and induce autoimmunity [44], the generation of Th17 cells during *Schistosoma japonicum* infection in C57BL/6 mice has determined to induce suppressive immunity to schistosome infection [48]. Interestingly, some Foxp3^+^ Treg cells could convert to IL-17^+^ T cells upon co-culture with dendritic cells selectively activated by dectin-1, a C-type lectin receptor involved in fungal recognition [49]. The conversion of Treg cells into Th17 cells may help restrain infections with specific fungi or other pathogens [50]. The flexibility between induced regulatory T cells and Th17 cells may affect the differentiation of CD4^+^ T cells and therefore may alter the direction of immune response [44, 51]. However, the relationship between the Treg and Th17 responses in *T. spiralis* infection remains unclear.

Together with our previous studies, our results further suggest the immunomodulatory function of *T. spiralis* paramyosin, which interacts with dendritic cells and stimulates regulatory T cells and Th17 cells. The data further support that *Ts*Pmy plays an important role in the immunomodulation of host immune response as a survival strategy, and is therefore a good candidate for vaccine development against trichinellosis. It is also possible to use r*Ts*Pmy as a therapeutic reagent for autoimmune or allergic diseases by taking advantage of its stimulating regulatory network of the immune system.

## Conclusions

Our results showed that *Ts*Pmy is able to activate mouse bone marrow-derived DCs to semi-mature status characterized by expressing CD40 and CD86, without CD80 and MHCII on the surface of CD11c^+^ DCs. The semi-matured *Ts*Pmy-pulsed DCs were able to stimulate *T. spiralis*-sensitized CD4^+^ T cells to proliferate associated with the secretion of IL-10 and TGF-β produced mostly by Treg cells. Incubation of *Ts*Pmy-pulsed DCs with naïve CD4^+^ splenocytes polarized the latter to CD4^+^CD25^+^Foxp3^+^ Tregs. However, mice immunized with r*Ts*Pmy only induce the CD4^+^CD25^−^Foxp3^+^ T cell population, associated with high level of IL-10 and TGF-β. r*Ts*Pmy also induced Th17 response, possibly converted from Foxp3^+^ Tregs. During *T. spiralis* infection, *Ts*Pmy plays an important role in modulating the host immune system by stimulating DCs to promote differentiation of regulatory T cells, in addition to binding to components of the host complement cascade, as survival strategies to live in host.

## Abbreviations

BMDCs: Mouse bone marrow-derived DC cells
CFSE: 5- and 6-carboxyfluorescein diacetate succinimidyl ester
Con-A: Concavalin-A
DCs: Dendritic cells
ELISA: Enzyme-linked immunosorbent assay
FACS: Fluorescence-activated cell sorting
IACUC: Institutional Animal Care and Use Committee
LPS: Lipopolysaccharide
mAb: Monoclonal antibody
MACS: Magnetic-activated cell sorting
MHCII: Major histocompatibility complex II
ML: Muscle larvae
PMA: Phorbol-12-myristate-13-acetate
PRRs: Pattern recognition receptors
r*Ts*Pmy: Recombinant *Ts*Pmy
Tregs: Regulatory T cells
*Ts*Pmy: *Trichinella spiralis* paramyosin
